# Quantitative MRI T2 Mapping is Able to Assess Tissue Quality After Reparative and Regenerative Treatments of Osteochondral Lesions of the Talus

**DOI:** 10.1002/jmri.27754

**Published:** 2021-05-28

**Authors:** Giulio Rizzo, Alessandro Cristoforetti, Alessandro Marinetti, Marta Rigoni, Leonardo Puddu, Fabrizio Cortese, Giandomenico Nollo, Sabino W. Della Sala, Francesco Tessarolo

**Affiliations:** ^1^ Division of Diagnostic Radiology Rovereto Hospital, Azienda Provinciale per i Servizi Sanitari Trento Italy; ^2^ Department of Industrial Engineering University of Trento Trento Italy; ^3^ Healthcare Research and Innovation Program (IRCS‐FBK‐PAT) Bruno Kessler Foundation Trento Italy; ^4^ Division of Orthopaedics and Traumatology Rovereto Hospital, Azienda Provinciale per i Servizi Sanitari Trento Italy

**Keywords:** articular cartilage, ankle, T2 relaxation time

## Abstract

**Background:**

Quantitative MRI has potential for tissue characterization after reparative and regenerative surgical treatment of osteochondral lesions of the talus (OCLTs). However available data is inconclusive and quantitative sequences can be difficult to implement in real‐time clinical application.

**Purpose:**

To assess the potential of T2 mapping in discriminating articular tissue characteristics after reparative and regenerative surgery of OCLTs in real‐world clinical settings.

**Study Type:**

Observational and prospective cohort study.

**Population:**

15 OCLT patients who had received either reparative treatment with arthroscopic microfracture surgery (MFS) for a grade I lesion or regenerative treatment with bone marrow derived cell transplantation (BMDCT) for a grade II lesion.

**Field Strength/Sequence:**

1.5 T, proton density weighted TSE, T2‐weighted true fast imaging with steady‐state‐free precession and multi‐echo T2 mapping sequences.

**Assessment:**

Patients were evaluated at a minimum postoperative follow‐up of 24 months. T2 maps of the ankle were generated and the distribution of T2 values was analyzed in manually identified volumes of interest (VOIs) for both treated lesions (TX) and healthy cartilage (CTRL). The amount of fibrocartilage, hyaline‐like and remodeling tissue in TX VOIs was obtained, based on T2 thresholds from CTRL VOIs.

**Statistical Tests:**

Fisher's exact test for categorical data, nonparametric Mann–Whitney *U* test for continuous data. The statistical significance level was *P* < 0.05.

**Results:**

From CTRL VOI analysis, T2 < 25 msec, 25 msec ≤ T2 ≤ 45 msec, and T2 > 45 msec were considered as representative for fibrocartilage, hyaline‐like and remodeling tissue, respectively. Tissue composition of the two treatment groups was different, with significantly more fibrocartilage (+28%) and less hyaline‐like tissue (−15%) in MFS than in BMDCT treated lesions. No difference in healthy tissue composition was found between the two groups (*P* = 0.75).

**Data Conclusions:**

T2 mapping of surgically treated OCLTs can provide quantitative information about the type and amount of newly formed tissue at the lesion site, thereby facilitating surgical follow‐up in a real‐word clinical setting.

**Level of Evidence:**

2

**Technical Efficacy:**

Stage 3

Osteochondral lesions of the talus (OCLTs) are defects involving both cartilage tissue and subchondral bone. Most frequently, they are secondary to trauma with an estimated occurrence of 6% in all ankle sprains; a lower number is due to nontraumatic etiology.^1^, ^2^

Surgical treatment of OCLTs aims at restoring continuity of the articular surface and joint function, reducing pain, and preventing evolution to degenerative osteoarthritis.^3^ Among surgical options, reparative treatments are techniques that stimulate bone marrow through microfractures (MFS) or drilling, inducing clot formation that typically evolves into fibrocartilage.^4^, ^5^, ^6^, ^7^ Regenerative techniques are instead based on autologous chondrocytes implantation or transplantation of stem cells with chondrogenic potential and are expected to result in hyaline‐like tissue, with biological, chemical, and functional characteristics close to healthy cartilage.^8^, ^9^, ^10^, ^11^ Reparative techniques have shown good results from a clinical point of view in short and medium term,^12^ but divergent results at long‐term follow‐up.^6^, ^7^ For regenerative techniques, while histologic evidence of formation of hyaline‐like tissue is limited,^8^ it has been associated with better clinical scores.^10^ Therefore, regular follow‐up evaluation of surgical intervention is required. However, arthroscopic or histological assessment of the newly formed tissue after OCLT surgery is invasive and noninvasive imaging techniques should be considered.

MRI is able to provide quantitative information about tissue formed after OCLT surgery in a noninvasive way through T2 mapping sequences.^11^, ^13^ T2 values depend on collagen fiber network organization, water coordination and content.^13^ T2 mapping therefore has potential value for predicting long term clinical outcome.^10^

Unfortunately, MRI studies on OCLTs have inconsistent results, showing T2 values of tissues obtained after MFS equivalent to normal cartilage^7^ or higher.^14^ In addition, T2 mapping of OCLTs is typically performed with 3 T MRI scanners and time‐consuming sequences.^14^, ^15^ These technical aspects limit the applicability of the technique to research studies in large and specialized radiological centers.

Thus the aim of this study was to evaluate the potential of T2 mapping to discriminate tissue characteristics of newly formed tissue in OCLT after reparative and regenerative surgery in real‐world clinical settings, using a 1.5 T MRI scanner and a time‐effective protocol.

## Materials and Methods

The study was approved by the local Ethics Committee and patients gave written informed consent prior to participation.

### Study Group and MRI Protocol

The study considered for inclusion all patients surgically treated for symptomatic OCLTs at the Division of Orthopedics and Traumatology of the Rovereto Hospital, Italy between July 2014 and March 2017.

Patients received either a reparative treatment with arthroscopic microfracture surgery (MFS) for a grade I lesion (lesion depth <0.8 mm and area <1.5 cm^2^), or a regenerative treatment with bone marrow derived cell transplantation (BMDCT) in case of grade II lesion (lesion depth <0.8 mm and area >1.5 cm^2^).^8^ Inclusion criteria required a minimum postoperative follow‐up of 24 months, no patient contraindications to MRI examination, and no postsurgical traumas and interventions at the lesion site.

Presurgery collected data included patient's age, sex, body mass index (BMI), and clinical orthopedic evaluation of the ankle joint according to the American Orthopedic Foot and Ankle Society (AOFAS) score.^16^

**FIGURE 1:**
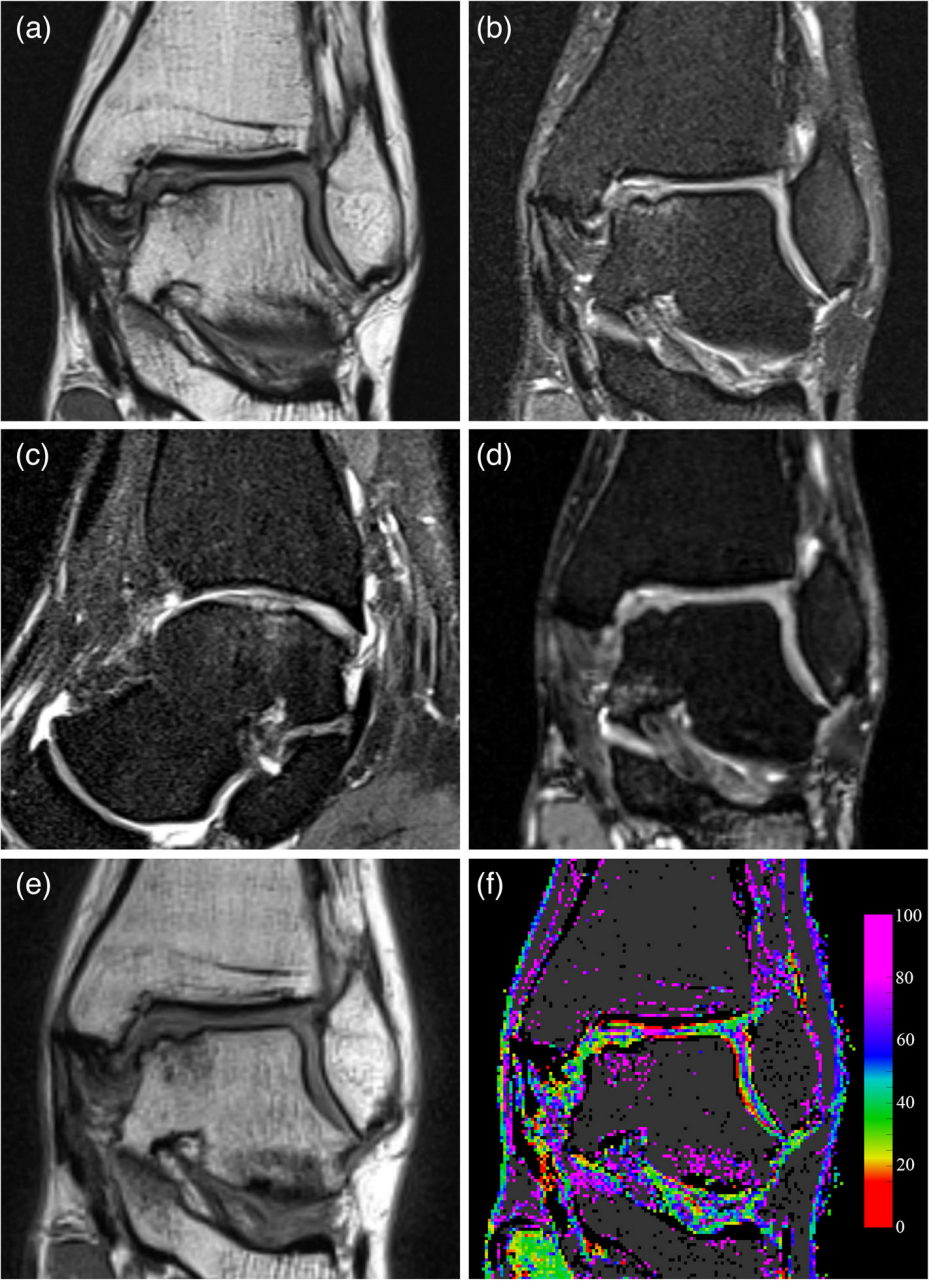
Mid‐term follow‐up MRI images of the talar bone region showing an OCLT. (a) Coronal proton density turbo spin‐echo (PD‐TSE). (b) Coronal fat suppressed PD‐TSE. (c) Sagittal fat suppressed PD‐TSE. (d) Coronal T2‐weighted true fast imaging with steady‐state‐free precession (T2‐TRUFI). (e) Coronal T2 multi‐echo sequence, first echo at TE = 13.8 msec. (f) Coronal T2 map obtained by processing the multi‐echo sequence, T2 values are represented according to the color map on the left. Voxels where curve fitting was unreliable or presenting low signal are shown in black, while possible bone tissue is shown in dark grey. The image was produced without interpolating color between voxels.

After a minimum of 24 months from surgery, patients were clinically re‐evaluated by the orthopedic surgeon, collecting AOFAS score at follow‐up. The following MRI sequences were acquired in a 1.5 T scanner (Magnetom Aera, Siemens Medical Systems Erlangen, Germany) using a Head/Neck 20 coil (Siemens Medical Systems Erlangen, Germany): (a) coronal proton density weighted turbo spin echo (PD‐TSE) with and without fat suppression (FS); repetition time (TR) 3150 msec and 3050 msec, respectively, echo time (TE) 39 msec, field of view (FOV) 170 mm × 170 mm, matrix size 384 × 384, pixel spacing (PS) 0.443 mm, slice thickness (ST) 2 mm, slice spacing (SS) 2 mm; (b) sagittal PD‐TSE FS; TR 2950, TE 32 msec, FOV 170 mm × 170 mm, matrix size 384 × 384, PS 0.443 mm, ST 2 mm, SS 2 mm; (c) coronal T2‐weighted true fast imaging with steady‐state‐free precession (T2‐TRUFI); TR 10.43 msec, TE 4.61 msec, flip angle 28°, FOV 170 mm × 170 mm, matrix size 256 × 256, PS 0.664 mm, ST 0.7 mm, SS 0.7 mm; and (d) coronal multi‐echo (5 echo train) and multi‐slice (26 slices) T2 mapping; TR 1000 msec, TE from 13.8 to 69 msec, FOV 170 mm × 170 mm, matrix size 256 × 256, PS 0.664 mm, ST 2 mm, SS 2 mm. Examples of the scan sequences for a representative patient are shown in Fig. 1a–e.

Based on the images from sequences (a), (b), and (c), a Magnetic resonance Observation of Cartilage Repair Tissue (MOCART) score^17^ was obtained at follow‐up. MOCART score was assigned by two radiologists (Alessandro Marinetti and Giulio Rizzo having, respectively, 20 and 4 years of clinical experience) following a consensus process. The opinion of a third radiologist (Sabino W. D. Sala, 40 years of experience in musculoskeletal radiology) was asked for debated cases. In addition, the multi‐echo images collected with sequence d), were processed to obtain T2 relaxation maps of the ankle for characterization of the articular tissue.

### Generation of T2 Maps

Multi‐echo images were processed by a custom‐realized software developed in the MATLAB programming platform (The MathWorks, Inc. Natick, MA, USA). Relaxation times in each voxel were obtained from the signal intensities $M_{i}$ at the different echo times ${\mathrm{TE}}_{i}$ (*i* = 1, …, 5), employing a noise bias correction scheme based on the methods of McGibney and Smith^18^ and of Miller and Joseph.^19^ Assuming a Rician noise distribution,^20^ the unbiased estimate of the power signal $P_{i}=M_{i}^{2}-2\sigma_{\text{rice}}^{2}$ was computed, where the Rice noise variance *σ*_rice_ was estimated as the second‐order moment in the background (air) region of all the image stacks: σ2=110∑i=15Mi2back. The background region was automatically segmented by the software. The model for the power signal decay P^i=A02exp−2TEiT2 was fitted on the $P_{i}$ sequence, finding the optimal *A*_0_ and T2 values that minimized the weighted sum of square of errors.

In order to assess the quality of fitting, the biased estimate of the echo signal was reconstructed as ${\hat{M}}_{i}=\sqrt{{\hat{P}}_{i}+2\sigma_{\text{rice}}^{2}}$, and the following parameters were computed: the signal to noise ratio SNR=15σrice2∑i=15Mi2, the coefficient of determination $R_{2}=1-\frac{\sum_{i=1}^{5}{\left(M_{i}-{\hat{M}}_{i}\right)}^{2}}{\sum_{i=1}^{5}{\left(M_{i}-\bar{M}\right)}^{2}}$, where M¯=15∑i=15Mi, and the reduced χ2=13σrice2∑i=15Mi−M^i2. Voxels having SNR<5 or $R_{2}<0.5$ or $\chi^{2}>10$ were excluded from the analysis. Based on previous experience on cartilage T2 mapping, only voxels with T2 values within the 5–100 msec range were considered as representative of articular tissue. The following criteria were also applied to exclude voxels not representative of articular tissue: ${\hat{M}}_{1}<200$ (low signal intensity at the first echo time) and A0exp−80T2>300 (high‐signal intensity at long echo time, typical for bone tissue). An example of T2 map with voxels filtered according to the above reported criteria is shown in Fig. 1f.

### Volume of Interest Segmentation

**FIGURE 2:**
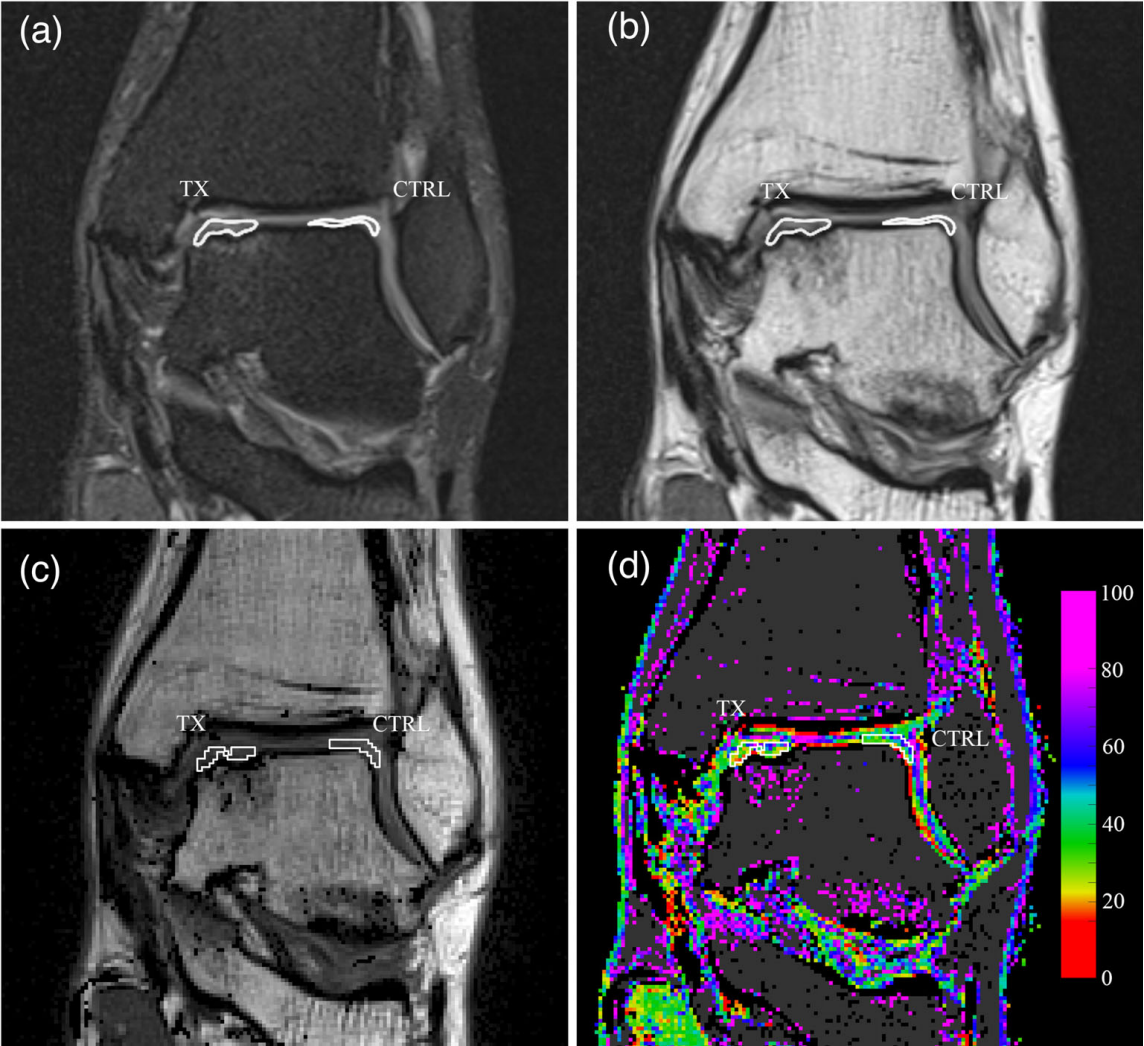
Definition and transposition of the volumes of interest (VOIs). (a) Manual delineation of the surgically treated area (TX) and cartilage control area (CTRL), on the coronal PD‐TSE sequence performed in Horos software. The VOIs are shown as contour lines on the interpolated rendering of the image. (b) The same VOIs in the first echo signal of the multi‐echo sequence, after optional alignment correction. (c) The VOIs in the first echo signal imported in MATLAB software. VOIs are delineated in white. (d) The same VOIs transposed on the T2 map, also shown in white. The image was produced without interpolating color between voxels.

The coronal MRI morphological images of the PD‐TSE sequences were processed by a radiologist (Giulio Rizzo, 4‐year clinical experience) supervised by a second radiologist (Alessandro Marinetti, 20 years of clinical experience) both with a specialty in musculoskeletal MRI, using the software Horos (Purview, Annapolis, MD, USA). Every segmentation was checked by the second radiologist and changes were made following a consensus process. Two volumes of interest (VOIs) for each ankle (Fig. 2a,b) were delineated: the treated lesion (TX) VOI, including the newly formed tissue at the treatment site, and the control (CTRL) VOI, identified within the healthy cartilage on the talar dome opposite to the treatment site. Each VOI was segmented in the PD‐TSE sequence, carefully excluding the subchondral bone and the synovial fluid. The absence of patient motion between the PD‐TSE and the first echo signal of the multi‐echo sequence was visually checked and manual registration of the VOI in the multi‐echo sequence was provided if necessary. Finally, the VOIs were imported into the MATLAB software and automatically superimposed to the T2 maps (Fig. 2c,d).

### Data Analysis

To identify the most reliable T2 intervals for hyaline cartilage, a first analysis focused on the T2 values of voxels in the CTRL VOIs was performed. The first quartile (Ta) and third quartile (Tb) of the pooled T2 values from all CTRL VOIs were calculated and considered, respectively, as the minimum and the maximum T2 values for hyaline/hyaline‐like tissue. T2 values lower than Ta were considered representative of fibrocartilage; values higher than Tb were considered representative of tissue under remodeling.^9^ To check homogeneity of the healthy cartilage in the two treatment groups, first and third quartiles were also calculated separately for the pooled T2 values distribution in CTRL VOIs in the MFS treated patients and in the BMDCT treated patients.

**FIGURE 3:**
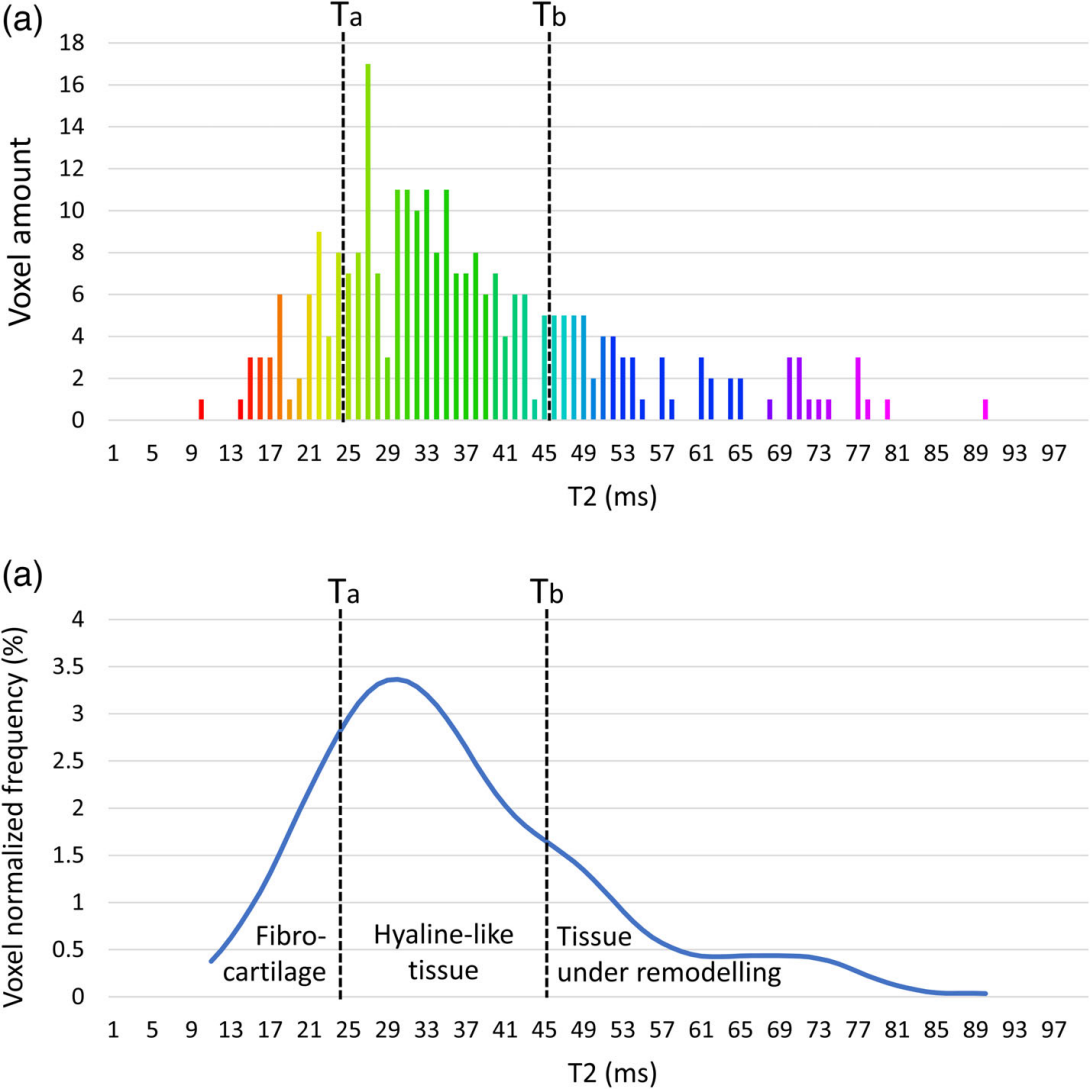
Example of the analysis of T2 data from voxels in a single volume of interest (VOI). (a) Frequency histogram showing the number of voxels according to their T2 values. Ta and Tb represent the first and third quartile of the cumulative T2 distribution of control VOIs. (b) Normalized frequency distribution of the same T2 values obtained using a 10 msec moving average filter. The percentage of fibrocartilage, hyaline‐like and remodeling tissue in the VOI was quantified by calculating the percentage of voxels having T2 values respectively lower than Ta, between Ta and Tb, and higher than Tb. The same percentages correspond to the three areas under the normalized distribution curve.

The analysis of the T2 data for VOIs was then performed in three steps. First, the frequency histogram showing the number of voxels according to their T2 values was realized for each patient, for both CTRL and TX VOIs (Fig. 3a). Second, the normalized frequency distribution of T2 values in each VOI was calculated using a 10 msec moving average (Fig. 3b). This allowed comparison of the T2 distributions between VOIs of different size. Third, the percentage of fibrocartilage, hyaline‐like and remodeling tissue in each VOI was quantified by calculating the percentage of voxels having T2 values respectively lower than Ta, between Ta and Tb, and higher than Tb.

### Statistics

Descriptive patients' and OCLTs' variables were expressed by first, second and third quartiles of their distributions. Dichotomous variables or scores were expressed as frequencies and percentages of occurrence.

Variables were analyzed based on treatment received by patients (i.e. BMDCT and MFS). Fisher's exact test was used to compare categorical data. Continuous data were checked for normality using Shapiro–Wilk test and nonparametric Mann–Whitney *U* test was used to compare nonnormally distributed data. A *P*‐value <0.05 was considered to be statistically significant. Statistical analyses were performed using the Stata software, (StataCorp, College Station, Texas USA).

## Results

### Study Groups and Tissue Evaluations

The study considered a total of 35 patients (14 MFS and 21 BMDCT). Five MFS and 10 BMDCT patients declined the invitation for the follow up MRI. Two cases (1 MFS and 1 BMDCT) were excluded because they received surgical treatment different from the one defined in the inclusion criteria. Two MFS and one BMDCT patient were excluded after the MRI examination because of presenting a postsurgical osteoarthritis with residual cartilage thickness insufficient to exclude the subchondral bone and the synovial fluid in VOIs delineation. The final study group consisted of 15 cases (11 males and 4 females, mean age at surgery 35 ± 14 years, mean age at follow‐up 39 ± 14 years) including six patients treated with MFS and nine patients with BMDCT. Patients' characteristics, OCLTs' details, clinical and imaging scores are summarized in Table 1. The two treatment groups showed no significant differences in terms of patients' characteristics, apart from lesion size and grading.

**TABLE 1:** Patients' and OCLTs' Characteristics According to Regenerative (BMDCT) and Reparative (MFS) Treatments

Patients' Data	BMDCT	MFS	*P*‐Value
Patients, *N* (%)	9 (100)	6 (100)	
Male, *N* (%)	7 (78)	4 (67)	1.00 *
Female, *N* (%)	2 (22)	2 (33)	
Body mass index (kg/cm^2^), median (q1–q3)	27 (23–29)	24 (22–25)	0.43 **
Age at surgery (years), median (q1–q3)	36 (27–44)	28 (20–51)	0.52 **
Age at follow‐up (years), median (q1–q3)	40 (31–48)	32 (23–56)	0.55 **
Follow‐up (months), median (q1–q3)	47 (42–50)	48 (38–57)	0.77 **
Lesions characteristics			
Lateral defect, *N* (%)	4 (44)	3 (50)	1.00 *
Medial defect, *N* (%)	5 (56)	3 (50)	
Left side, *N* (%)	4 (44)	3 (50)	1.00 *
Right side, *N* (%)	5 (56)	3 (50)	
Defect's surface area (cm^2^), median (q1–q3)	1.00 (0.87–1.16)	0.30 (0.27–0.73)	0.02 **
Defect's depth (mm), median (q1–q3)	5 (5–7)	5 (5–7)	0.90 **
Clinical scores			
AOFAS presurgery, median (q1–q3)	72 (72–76)	79 (73–79)	0.06 **
AOFAS at follow‐up, median (q1–q3)	96 (90–100)	100 (100–100)	0.18 **
AOFAS score improvement (%), median (q1–q3)	90 (62–100)	100 (100–100)	0.18 **
Imaging scores			
MOCART at follow‐up, median (q1–q3)	55 (50–60)	68 (65–75)	0.05 **

AOFAS = The American Orthopedic Foot and Ankle Society; BMDCT = bone marrow derived cells transplantation; MFS = microfracture; MOCART = Magnetic Resonance Observation of Cartilage Repair Tissue; q1 = first quartile; q3 = third quartile.

* Fisher's exact test.

** Nonparametric Mann–Whitney *U* test.

On average, 193 and 173 voxels were identified in each CTRL VOI and TX VOI, respectively, corresponding to a tissue volume of 170 mm^3^ and 152 mm^3^. About 8% of these voxels were excluded according to the criteria defined in the methods section leaving a total of 2674 and 2416 voxels, over the 15 patients, for the CTRL and TX VOIs, respectively.

The analysis of the T2 cumulative distribution of all CTRL VOIs showed a median (first quartile–third quartile) of 35 (25–45) msec. Similar figures were obtained for the two treatment subgroups individually (34 (25–45) msec and 35 (25–46) msec in MFS (931 voxels) and BCMDT (1743 voxels) patients, respectively), confirming the homogeneity of the sub‐groups in term of healthy cartilage. Ta and Tb values were therefore set to 25 msec and 45 msec, respectively. Accordingly, the intervals T2 < 25 msec, 25 msec ≤ T2 ≤ 45 msec, and T2 > 45 were considered to be representative of fibrocartilage, hyaline‐like tissue and tissue undergoing remodeling, respectively.

**FIGURE 4:**
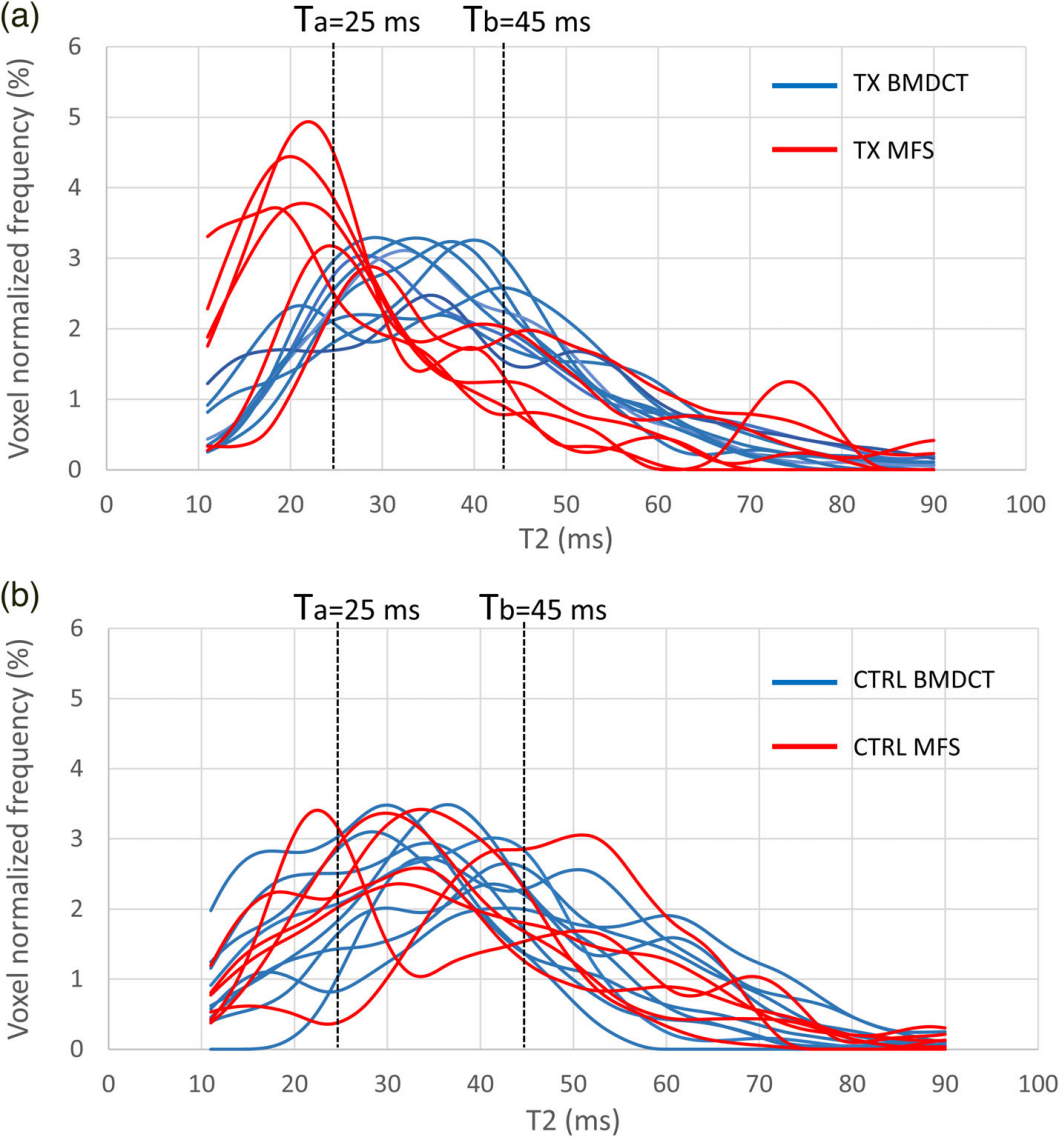
Normalized frequency histograms of T2 values of each volume of interest (VOI) in the whole study population. (a) Treated (TX) VOIs. (b) Control (CTRL) VOIs. Data in each panel are color‐coded according to the treatment type (MFX in red, BMDCT in blue). Dashed lines indicate T2 values of Ta = 25 msec and Tb = 45 msec considered as boundaries for differentiating between fibro‐cartilage (T < 25 msec), hyaline‐like tissue (25 msec < T2 < 45 msec), and tissue under remodeling (T > 45 msec).

**FIGURE 5:**
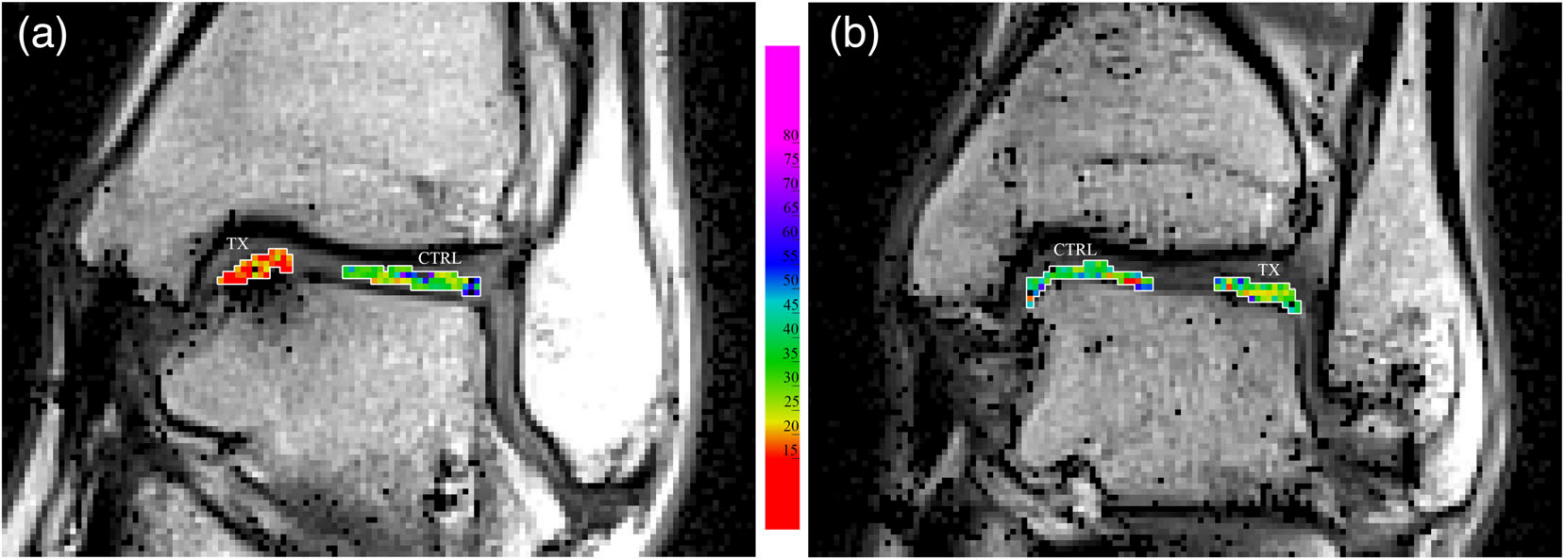
T2 values inside the treatment (TX) and control (CTRL) VOIs, delineated in white, superimposed on the image of first echo signal. (a) Patient treated with MFS. (b) Patient treated with BMDCT. Images were produced without interpolating color between voxels.

The normalized frequency distributions of the T2 values in the TX and CTRL VOIs of all patients, are shown in Fig. 4. Lesion sites (TX VOIs) distributions for MFS treated lesions were peaked at lower T2 values than BMDCT (Fig. 4a). This difference was not present in the corresponding CTRL VOIs (Fig. 4b). Consistently, T2 maps in Fig. 5a showed that MFS treated areas were mainly composed of voxels in the yellow‐red color range (T2 < 25 msec, fibrous tissue). On the contrary, BMDCT treated areas showed mainly voxels in the green color range (25 msec ≤ T2 ≤ 45 msec, hyaline‐like tissue), similar to voxels in CTRL areas of both treatment groups (Fig. 5b).

**FIGURE 6:**
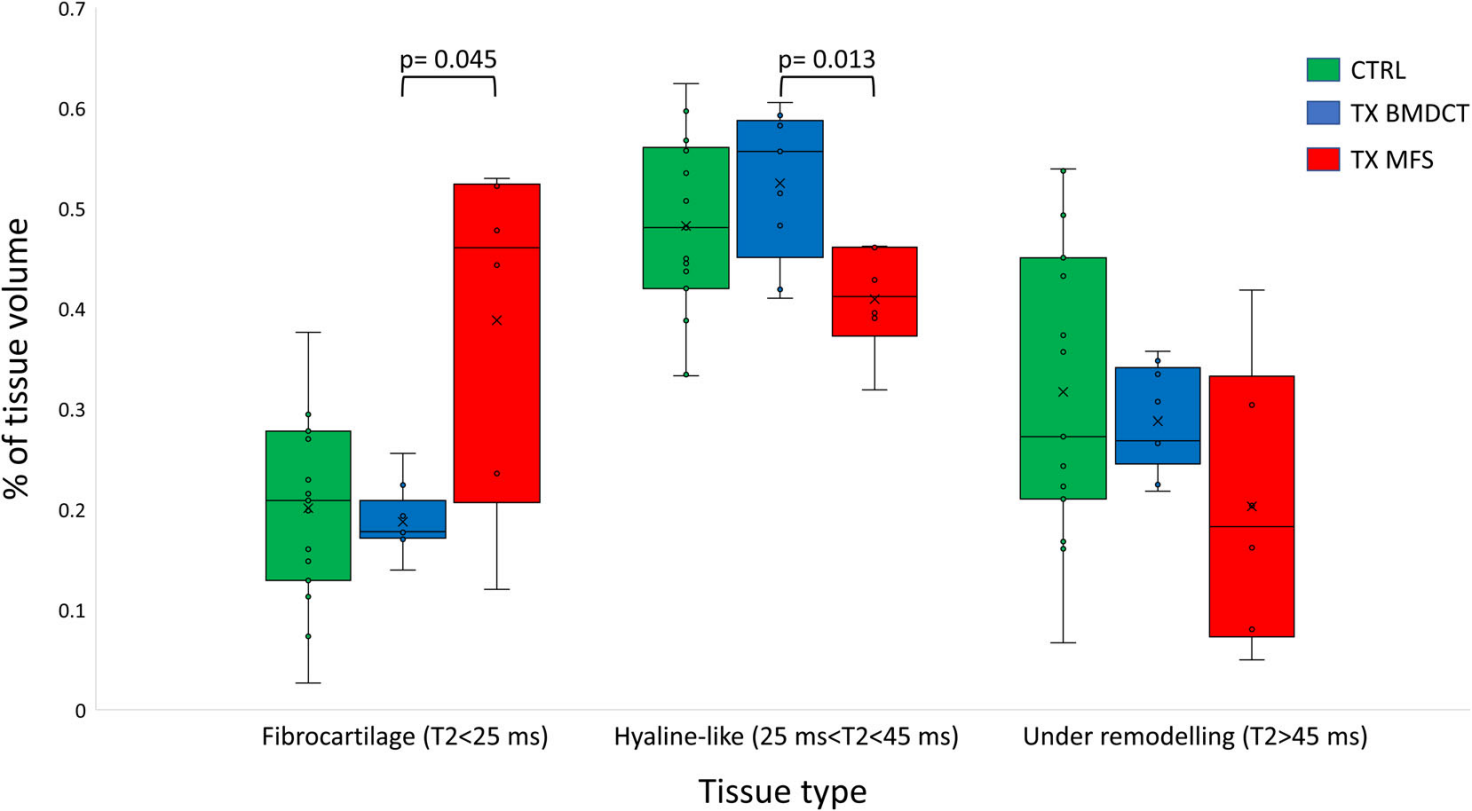
Box plot summarizing the percentage of the three different tissue types evaluated by T2 mapping in different volumes of interests: controls (*N* = 15) (CTRL), lesions treated according to regenerative surgery (*N* = 9) (TX BMDCT), and lesions treated according to reparative surgery (*N* = 6) (TX MFS).

The amount of the three different tissue types in TX areas is reported as percentage of VOI volume in Table 2. The comparison of tissue type percentages between the two treatment groups is presented in Fig. 6. Significant differences were found in tissue composition between the two treatment groups, having a higher percentage of fibrocartilage and a lower percentage of hyaline‐like tissue in MFS than in BMDCT treated lesions. The corresponding analysis of the CTRL VOIs showed no statistical differences in healthy tissue composition between MFS and BMDCT treated patients (*P* = 0.724, *P* = 0.724, *P* = 0.814 for T2 < 25 msec, 25 msec < T2 < 45 msec, and T2 > 45 msec, respectively), confirming the homogeneity of control cartilage in patients of the two treatment groups.

**TABLE 2:** Percentages of Newly Formed Tissue Type at Lesion Site (TX) and Control VOIs (CTRL) According to Regenerative (BMDCT) and Reparative (MFS) Surgical Treatment^†^

		Treatment Subgroups		
Patient Group	Tissue Type (T2 Range)	BMDCT (%), median (q1–q3)	MFS (%), median (q1–q3)	*P*‐Value*
Treated (TX)	Volume of fibrous tissue (T2 < 25 msec)	0.18 (0.17–0.19)	0.46 (0.24–0.52)	0.045
	Volume of hyaline‐like tissue (25 msec < T2 < 45 msec)	0.56 (0.48–0.58)	0.41 (0.39–0.46)	0.013
	Volume of remodeling tissue (T2 > 45 msec)	0.27 (0.27–0.33)	0.18 (0.08–0.30)	0.126
Controls (CTRL)	Volume of fibrous tissue (T2 < 25 msec)	0.16 (0.13–0.27)	0.21 (0.20–0.28)	0.724
	Volume of hyaline‐like tissue (25 msec < T2 < 45 msec	0.51 (0.44–0.56)	0.45 (0.39–0.57)	0.724
	Volume of remodeling tissue (T2 > 45 msec)	0.24 (0.21–0.45)	0.31 (0.22–0.37)	0.814

BMDCT = bone marrow derived cells transplantation; MFS = microfracture.

* Nonparametric Mann–Whitney *U* test.

^†^ Statistical significance of differences between the two treatment subgroups is also reported.

## Discussion

MRI has prognostic potential by noninvasively characterizing newly formed tissue at the lesion site.^11^, ^13^ A range of compositional MRI techniques have been proposed^13^ to infer biochemical composition and mechanical properties^21^ of the chondral tissue. However, many of these techniques are still limited to research settings and clinical trials, requiring further validation and dedicated equipment.^22^, ^23^

Among the available techniques, T2 mapping is a well‐established quantitative approach, sensitive to collagen fiber network organization and water content.^11^, ^24^ Most T2 studies on knee cartilage have reported lower T2 values for MFS induced fibrocartilage with respect to hyaline‐like tissue,^25^, ^26^ but literature on the talus region is not definitive.^7^, ^15^, ^27^ T2 relaxation time in the newly formed tissue depends on the time from treatment, with initial higher values associated with tissue under remodeling, and subsequent lower and more stable values being reported after a maturation period of 1–2 years.^28^ Considering these aspects, this study included patients with a minimum follow‐up of 24 months and investigated the ability of T2 mapping to discriminate newly formed tissue at surgically treated OCLTs. The study was performed using a time‐effective sequence (<5 min) in a 1.5 T scanner which could realistically be used in a clinical setting. Such “real‐world” data enhances the efficiency of research, bridging the gap between clinical research and practice, and increases the transfer of research findings into clinical settings.^23^, ^29^

In this study, the T2 thresholds that were used to define different tissue types were tailored to the healthy tissue of the same study population. The T2 range for the control cartilage in this study (25–45 msec) fell within the range of values reported by previous studies on healthy ankle cartilage: 37 ± 11 msec,^15^ 37 ± 7 msec,^30^ 39 ± 8 msec,^31^ 36 ± 4 msec,^32^ and 47 ± 9 msec.^33^ Minor variability in observed healthy cartilage T2 values can be due to differences in scanners, acquisition protocols, analysis methodology, and segmentation.^11^ The use of internal controls could help in reducing variability and increasing data reliability. Similar T2 values for healthy cartilage in the two treatment subgroups (Table 1) were found in this study, thus adding robustness to the results obtained from the comparison of TX VOIs. A markedly different shape of the T2 distributions emerged when comparing TX VOIs according to treatment subgroups. A higher percentage of fibrocartilage and a lower percentage of hyaline‐like tissue in MFS than in BMDCT treated lesions was observed. These results were consistent with a higher content of densely packed type I collagen fibers having less interstitial water of fibrocartilage, as previously shown with a higher field scanner.^27^ In addition, data from BMDCT treated lesions were in line with several studies^9^, ^10^, ^34^ indicating that regenerated cartilage was similar to hyaline cartilage in terms of T2 relaxation time.

Cartilage classification by T2 intervals alone does not represent a conclusive tissue characterization, since while low T2 may typically indicate fibrocartilage, it may also indicate cartilage desiccation. Likewise, high T2 values are associated with both remodeling and osteoarthritic tissue.^35^

Previous data on ankle cartilage^9^, ^10^, ^34^ were obtained with dedicated coils and/or 3 T scanners. In our study, the feasibility of T2 mapping on newly formed tissue of OCLTs was confirmed in the clinical setting, and new data on T2 values for tissues resulting from both regenerative and reparative surgical techniques was generated. The method presented here could be applied at different time points from surgery, potentially allowing for a comparative assessment of the treated site at follow‐up. This approach paves the way to a real‐world clinical use of noninvasive T2 analysis of talus cartilage, complementing morphological imaging in the assessment of OCLT treatment outcome.

### Limitations

The absence of second‐look arthroscopy limited the possibility of validating tissue characterization based on T2. However, the comparative design of the study and the use of internal controls added confidence to the MRI findings. The use of an internal control for healthy cartilage within the same lesioned ankle could introduce biased T2 values due to biomechanics alteration of the treated ankle.^32^ However, several advantages are associated with this study design and the same approach has been previously applied in several studies,^15^, ^31^ whereas only few have opted for an external control group.^9^, ^27^

From a technical perspective, the application of a time effective sequence in a 1,5 T scanner implied acquisitions with reduced spatial resolution and increased partial volume effects, which would result in more difficulties in delineating the VOIs, and insufficient resolution to explore differences in cartilage thickness.^27^

Another limitation was the small population of this pilot study. While this was sufficient to evidence a statistical difference in T2 between the two treatment subgroups, further analyses (eg, correlation between T2 and clinical or imaging scores) are needed in larger cohorts of patients.^10^, ^15^

A further limitation is that cartilage T2 values may have regional variations, as detected with a 3 T scanner,^36^ which may increase the analysis error. Since both medial and lateral surgical sites were present in our datasets, the T2 values we reported for the different tissue types are also affected by this error.

Finally, the analysis reported here was focused on the articular tissue. Future studies should take into account bone‐cartilage cross‐talk^37^ in OCL, in order to provide a more comprehensive evaluation of the whole osteochondral unit.

## Conclusion

T2 mapping of surgically treated OCLTs is a promising tool to provide quantitative information about the type and relative amount of fibrocartilage and hyaline‐like tissue formed at the lesion site. Our preliminary results suggest the feasibility of obtaining this information by statistical analysis of local voxels in a real‐world clinical setting with a 1.5 T scanner and time effective sequences.

## References

[1] Elias I , Zoga AC , Morrison WB , et al. Osteochondral lesions of the talus: Localization and morphologic data from 424 patients using a novel anatomical grid scheme. Foot Ankle Int 2007;28:154‐161. 10.3113/FAI.2007.0154. 17296131

[2] Looze CA , Capo J , Ryan MK , et al. Evaluation and management of osteochondral lesions of the talus. Cartilage 2017;8:19‐30. 10.1177/1947603516670708. 27994717 PMC5154424

[3] van Dijk CN , Reilingh ML , Zengerink M , van Bergen CJA . Osteochondral defects in the ankle: Why painful? Knee Surg Sports Traumatol Arthrosc 2010;18:570‐580. 10.1007/s00167-010-1064-x. 20151110 PMC2855020

[4] Murawski CD , Kennedy JG . Operative treatment of osteochondral lesions of the talus. J Bone Joint Surg Am 2013;95:1045‐1054. 10.2106/JBJS.L.00773. 23780543

[5] Lee K‐B , Bai L‐B , Yoon T‐R , et al. Second‐look arthroscopic findings and clinical outcomes after microfracture for osteochondral lesions of the talus. Am J Sports Med 2009;37 (Suppl. 1):63S‐70S. 10.1177/0363546509348471. 19843658

[6] Ferkel RD , Zanotti RM , Komenda GA , et al. Arthroscopic treatment of chronic osteochondral lesions of the talus: Long‐term results. Am J Sports Med 2008;36:1750‐1762. 10.1177/0363546508316773. 18753679

[7] Becher C , Zühlke D , Plaas C , et al. T2‐mapping at 3 T after microfracture in the treatment of osteochondral defects of the talus at an average follow‐up of 8 years. Knee Surg Sports Traumatol Arthrosc 2015;23:2406‐2412. 10.1007/s00167-014-2913-9. 24562698

[8] Giannini S , Buda R , Faldini C , et al. Surgical treatment of osteochondral lesions of the talus in young active patients. J Bone Joint Surg Am 2005;87 (Suppl. 2):28‐41. 10.2106/JBJS.E.00516. 16326721

[9] Giannini S , Battaglia M , Buda R , et al. Surgical treatment of osteochondral lesions of the talus by open‐field autologous chondrocyte implantation: A 10‐year follow‐up clinical and magnetic resonance imaging T2‐mapping evaluation. Am J Sports Med 2009;37 (Suppl. 1):112S‐118S. 10.1177/0363546509349928. 19934440

[10] Pagliazzi G , Vannini F , Battaglia M , et al. Autologous chondrocyte implantation for talar osteochondral lesions: Comparison between 5‐year follow‐up magnetic resonance imaging findings and 7‐year follow‐up clinical results. J Foot Ankle Surg 2018;57:221‐225. 10.1053/j.jfas.2017.05.013. 29146220

[11] Surowiec RK , Lucas EP , Ho CP . Quantitative MRI in the evaluation of articular cartilage health: Reproducibility and variability with a focus on T2 mapping. Knee Surg Sports Traumatol Arthrosc 2014;22:1385‐1395. 10.1007/s00167-013-2714-6. 24170187

[12] Chuckpaiwong B , Berkson EM , Theodore GH . Microfracture for osteochondral lesions of the ankle: Outcome analysis and outcome predictors of 105 cases. Arthroscopy 2008;24:106‐112. 10.1016/j.arthro.2007.07.022. 18182210

[13] Martín Noguerol T , Raya JG , Wessell DE , et al. Functional MRI for evaluation of hyaline cartilage extracelullar matrix—A physiopathological‐based approach. Br J Radiol 2019;92:20190443. 10.1259/bjr.20190443. 31433668 PMC6849690

[14] Domayer SE , Welsch GH , Stelzeneder D , et al. Microfracture in the ankle: Clinical results and MRI with T2‐mapping at 3.0 T after 1 to 8 years. Cartilage 2011;2:73‐80. 10.1177/1947603510380901. 26069571 PMC4300787

[15] Rehnitz C , Kuni B , Wuennemann F , et al. Delayed gadolinium‐enhanced MRI of cartilage (dGEMRIC) and T2 mapping of talar osteochondral lesions: Indicators of clinical outcomes. J Magn Reson Imaging 2017;46:1601‐1610. 10.1002/jmri.25731. 28419612

[16] Kitaoka HB , Alexander IJ , Adelaar RS , et al. Clinical rating systems for the ankle‐hindfoot, midfoot, hallux, and lesser toes. Foot Ankle Int 1994;15:349‐353. 10.1177/107110079401500701. 7951968

[17] Marlovits S , Singer P , Zeller P , et al. Magnetic resonance observation of cartilage repair tissue (MOCART) for the evaluation of autologous chondrocyte transplantation: Determination of interobserver variability and correlation to clinical outcome after 2 years. Eur J Radiol 2006;57:16‐23. 10.1016/j.ejrad.2005.08.007. 16203119

[18] McGibney G , Smith MR . An unbiased signal‐to‐noise ratio measure for magnetic resonance images. Med Phys 1993;20:1077‐1078. 10.1118/1.597004. 8413015

[19] Miller AJ , Joseph PM . The use of power images to perform quantitative analysis on low SNR MR images. Magn Reson Imaging 1993;11:1051‐1056. 10.1016/0730-725x(93)90225-3. 8231670

[20] Gudbjartsson H , Patz S . The Rician distribution of noisy MRI data. Magn Reson Med 1995;34:910‐914. 10.1002/mrm.1910340618. 8598820 PMC2254141

[21] Truhn D , Sondern B , Oehrl S , et al. Differentiation of human cartilage degeneration by functional MRI mapping‐an ex vivo study. Eur Radiol 2019;29:6671‐6681. 10.1007/s00330-019-06283-9. 31187218

[22] Duarte A , Ruiz A , Ferizi U , et al. Diffusion tensor imaging of articular cartilage using a navigated radial imaging spin‐echo diffusion (RAISED) sequence. Eur Radiol 2019;29:2598‐2607. 10.1007/s00330-018-5780-9. 30382348 PMC6445706

[23] Link TM . Editorial comment: The future of compositional MRI for cartilage. Eur Radiol 2018;28:2872‐2873. 10.1007/s00330-018-5457-4. 29713777 PMC5988989

[24] Juras V , Zbýň Š , Mlynarik V , et al. The compositional difference between ankle and knee cartilage demonstrated by T2 mapping at 7 Tesla MR. Eur J Radiol 2016;85:771‐777. 10.1016/j.ejrad.2016.01.021. 26971422

[25] Welsch GH , Trattnig S , Domayer S , et al. Multimodal approach in the use of clinical scoring, morphological MRI and biochemical T2‐mapping and diffusion‐weighted imaging in their ability to assess differences between cartilage repair tissue after microfracture therapy and matrix‐associated autologous chondrocyte transplantation: A pilot study. Osteoarthr Cartil 2009;17:1219‐1227. 10.1016/j.joca.2009.03.018. 19409295

[26] Domayer SE , Kutscha‐Lissberg F , Welsch G , et al. T2 mapping in the knee after microfracture at 3.0 T: Correlation of global T2 values and clinical outcome—Preliminary results. Osteoarthr Cartil 2008;16:903‐908. 10.1016/j.joca.2007.11.014. 18203632

[27] Domayer SE , Apprich S , Stelzeneder D , et al. Cartilage repair of the ankle: First results of T2 mapping at 7.0 T after microfracture and matrix associated autologous cartilage transplantation. Osteoarthr Cartil 2012;20:829‐836. 10.1016/j.joca.2012.04.015. 22542632

[28] Jungmann PM , Baum T , Bauer JS , et al. Cartilage repair surgery: Outcome evaluation by using noninvasive cartilage biomarkers based on quantitative MRI techniques? Biomed Res Int 2014;2014:840170. 10.1155/2014/840170. 24877139 PMC4024422

[29] Corrigan‐Curay J , Sacks L , Woodcock J . Real‐world evidence and real‐world data for evaluating drug safety and effectiveness. JAMA 2018;320:867‐868. 10.1001/jama.2018.10136. 30105359

[30] Nehrer S , Domayer SE , Hirschfeld C , et al. Matrix‐associated and autologous chondrocyte transplantation in the ankle: Clinical and MRI follow‐up after 2 to 11 years. Cartilage 2011;2:81‐91. 10.1177/1947603510381095. 26069572 PMC4300785

[31] Kubosch EJ , Erdle B , Izadpanah K , et al. Clinical outcome and T2 assessment following autologous matrix‐induced chondrogenesis in osteochondral lesions of the talus. Int Orthop 2016;40:65‐71. 10.1007/s00264-015-2988-z. 26346373

[32] Lee S , Yoon YC , Kim JH . T2 mapping of the articular cartilage in the ankle: Correlation to the status of anterior talofibular ligament. Clin Radiol 2013;68:e355‐e361. 10.1016/j.crad.2013.01.023. 23537578

[33] Quirbach S , Trattnig S , Marlovits S , et al. Initial results of in vivo high‐resolution morphological and biochemical cartilage imaging of patients after matrix‐associated autologous chondrocyte transplantation (MACT) of the ankle. Skeletal Radiol 2009;38:751‐760. 10.1007/s00256-009-0682-1. 19296100

[34] Battaglia M , Rimondi E , Monti C , et al. Validity of T2 mapping in characterization of the regeneration tissue by bone marrow derived cell transplantation in osteochondral lesions of the ankle. Eur J Radiol 2011;80:e132‐e139. 10.1016/j.ejrad.2010.08.008. 20801594

[35] Li X , Benjamin Ma C , Link TM , et al. In vivo T(1rho) and T(2) mapping of articular cartilage in osteoarthritis of the knee using 3 T MRI. Osteoarthr Cartil 2007;15:789‐797. 10.1016/j.joca.2007.01.011. PMC2040334 17307365

[36] Lockard CA , Chang A , Shin RC , et al. Regional variation of ankle and hindfoot cartilage T2 mapping values at 3 T: A feasibility study. Eur J Radiol 2019;113:209‐216. 10.1016/j.ejrad.2019.02.011. 30927949

[37] Findlay DM , Kuliwaba JS . Bone‐cartilage crosstalk: A conversation for understanding osteoarthritis. Bone Res 2016;4:16028. 10.1038/boneres.2016.28. 27672480 PMC5028726

